# Nationwide Exposure of U.S. Working Dogs to the Chagas Disease Parasite, *Trypanosoma cruzi*

**DOI:** 10.4269/ajtmh.19-0582

**Published:** 2020-03-16

**Authors:** Alyssa C. Meyers, Julia C. Purnell, Megan M. Ellis, Lisa D. Auckland, Marvin Meinders, Sarah A. Hamer

**Affiliations:** 1 Department of Veterinary Integrative Biosciences, Texas A&M University, College Station, Texas;; 2 College of Veterinary Medicine and Biomedical Sciences, Colorado State University, Fort Collins, Colorado;; 3 Department of Homeland Security, Office of Health Affairs, Washington, District of Columbia

## Abstract

*Trypanosoma cruzi* is a zoonotic protozoan parasite vectored by triatomine insects that are endemic to the Americas, including the southern United States. Surveillance of domestic dogs for *T. cruzi* exposure allows for the determination of geographic regions of transmission that are relevant for human and animal health. The U.S. Department of Homeland Security (DHS) working dogs provide critical security and detection services across the country, and many train or work in the southern United States, where they are at risk for *T. cruzi* exposure. We sampled blood from 1,610 working dogs (predominantly Belgian Malinois, German shepherds, and Labrador retrievers) from six task forces (including the Transportation Security Administration, Customs and Border Protection, Secret Service, and more) and two canine training centers across 41 states from 2015 to 2018. Canine sera that were reactive on at least two independent serological assays were considered positive for anti–*T.-cruzi* antibodies. In addition, up to three independent polymerase chain reaction (PCR) assays were used to detect and type *T. cruzi* DNA. Overall seroprevalence was 7.5%, and four dogs (0.25%, *n* = 1,610) had detectable parasite DNA in the blood, comprising parasite discrete taxonomic units (DTUs) TcIV and a coinfection of TcI/TcIV. Dogs that worked within versus outside of the geographic range of established triatomines showed comparable seroprevalence (7.3% and 9.2%, respectively; *P* = 0.61). Determining the prevalence of *T. cruzi* in these working dogs and looking at spatially associated risk factors have practical implications for disease risk management and could assist with improved control measures to protect both animal and human health.

## INTRODUCTION

*Trypanosoma cruzi* is a protozoan parasite and the etiologic agent of Chagas disease. Chagas disease is a zoonotic disease that affects more than eight million people throughout the Americas and a diversity of domestic and wild animals.^1^ *Trypanosoma cruzi* infections in humans and animals may be asymptomatic or may be associated with debilitating acute or chronic cardiac disease, characterized by myocarditis, hepatomegaly, ascites, cardiac dilatation, or sudden death.^2,3^ There are currently no vaccinations, and anti-parasitic treatments are limited in humans and not approved for dogs in the United States.^1^

The Southern United States harbors an established enzootic cycle of *T. cruzi*, where the parasite is vectored by several triatomine species and infects a diversity of mammalian hosts including raccoons, opossums, and domestic dogs.^1,4,5^ Interactions between humans and triatomine vectors in the United States can occur after disruption of vertebrate host habitats, causing sylvatic vectors to look for new habitats. In addition, attraction to lights and poor housing structures can allow for invasion into human dwellings.^1,6–8^ This epidemiological setting contrasts with what is commonly found in Central and South America, where triatomines more commonly colonize homes, and dogs are recognized to play an important role as *T. cruzi* reservoirs.^9–12^ The role of domestic dogs in the *T. cruzi* transmission cycle in the United States is not completely understood, although an increasing number of studies demonstrate exposure of diverse dog populations in the south, especially Texas, with reported seroprevalence ranging from 3.6 to 57.6%.^13–20^

Infection with *T. cruzi* is more likely in dogs than in humans.^21,22^ This could be due to differences in behavior, including a dog’s affinity to consume insects, allowing for oral *T. cruzi* transmission,^16,23–26^ and that dogs more commonly sleep outside, increasing their contact with nocturnal peridomestic vectors.^14,17,27,28^ In South America, dogs have been used as sentinels of human disease risk,^11,29^ yet the degree to which infected dogs may signal human disease risk in the United States is not well understood. Understanding spatial risk factors associated with *T. cruzi*–infected dogs could be informative for vector control initiatives benefiting both veterinary and public health.

We conducted an epidemiological investigation of dogs infected with *T. cruzi* by studying a population of government-owned working dogs from across the United States. Our objectives were to 1) determine the seroprevalence of dogs across the United States, with a focus on comparing working dog populations that live within the triatomine vector range and outside the range and 2) use a comparative diagnostic approach and multiple independent testing platforms to compare the prevalence of antibody-positive dogs versus dogs with circulating parasite DNA. We hypothesize that the prevalence of *T. cruzi* exposure is higher within the range of triatomine vectors and that any seropositive dogs outside the range would be attributed to the dog’s travel history to an endemic region. As it pertains to the particular study population of working dogs, symptomatic *T. cruzi* infections may limit a dog’s ability to work with follow-on security consequences. With a better understanding of the distribution of *T. cruzi* infection in working dogs, we can determine risk factors for exposure and provide targeted interventions to populations most at risk.

## METHODS

### Ethics statement.

All canine samples were collected in adherence with animal use protocols approved by the Texas A&M University’s Institutional Animal Care and Use Committee on March 22, 2017 under the number 2015-0289. Written consent was received for each dog sampled from the handler.

### Study population: DHS working dogs.

The U.S. DHS owns more than 3,000 working dogs across the United States assigned to the following task forces: Federal Protection Services, U.S. Coast Guard, Secret Service, Transportation Security Administration (TSA), or two task forces within the Customs and Border Protection (CBP): Border Patrol or Port of Entry. Many of the dogs were bred in Europe, but some were purchased from vendors across the United States. Dogs receive approximately 3–6 months of training at one of four training facilities in Texas (2), Virginia, or Alabama, and specialize in various jobs such as explosives detection; track and trail; detection of humans, narcotics, currency, agricultural products; and search and rescue. After training, dogs are typically assigned to a specific task force and management area and have limited travel (with the exception of Secret Service dogs that travel both within and outside of the country). When dogs are off duty, they are either kenneled individually at their handlers’ residence or in a group kennel.

### Sample collection.

A cross-sectional study design was used to collect blood samples from DHS working dogs across the United States from March 2017 to May 2018. In addition, test results from CBP dogs we previously sampled in Texas and New Mexico in 2015–2016^19^ were included in the analysis unless they were resampled in 2017–2018 in which case, the more recent test results were used. Samples were collected in two ways: from field sampling in California, Arizona, and Texas and from submissions by the dog’s veterinarians across 41 states, Washington D.C., and the U.S. Virgin Islands, with a goal of sampling at least 50% of all DHS working dogs. Detailed instructions were provided to veterinarians during a dog’s routine veterinary visit. For both the field sampling and the sampling at veterinary clinics, a minimum of 5 mL of blood was collected by venipuncture and aliquoted into serum and ethylenediaminetetraacetic acid (EDTA) tubes. The sample criteria included dogs older than 6 months and on active duty or in training. Demographic information was collected on all dogs sampled including age, sex, breed, canine job, sleeping location (home or kennel, indoors/outdoors), station of duty, and address.

### Serologic and molecular testing.

After an aliquot of anticoagulated whole blood was taken, the blood tubes were spun and separated into serum, clot, plasma, and buffy coat and frozen at −20°C until analysis. Serum samples were screened for anti–*T. cruzi* antibodies by Chagas Stat-Pak^®^ (ChemBio Diagnostic Systems Inc., Medford, NY), a rapid immunochromatographic test designed for human use, using previously described methods.^19^ Tests were considered negative when no color developed and positive when a clear line developed. In addition, very faint bands that were not perceptible enough to be considered a clear positive, yet with some low level of color development to differentiate them from negative, were tracked as “inconclusive” and subjected to additional testing. All positive and inconclusive samples as determined by Stat-Pak^®^ plus 10% of the negatives were tested by the following tests: 1) The indirect fluorescent antibody (IFA) test performed by the Texas Veterinary Medical Diagnostic Laboratory (TVMDL, College Station, TX). Titer values of 20 or higher were considered positive as per the TVMDL standard protocol. 2) Trypanosoma Detect™ (InBios, International, Inc., Seattle, WA), a rapid immunochromatographic test designed for human use. Both immunochromatographic tests have been used for antibody detection in dogs, and both have shown high sensitivity and specificity when compared with IFA.^14,15,17,30^ If two or more of the three tests were positive, an individual was classified as seropositive. When inconclusive results (faint bands) were present, they were counted as a negative test result.

DNA was extracted from 250 µL of buffy coat or clot samples using E.N.Z.A. Tissue DNA kit (Omega Bio-Tek, Norcorss, GA). Samples were screened for *T. cruzi* DNA using the Cruzi 1/2 primer set and Cruzi 3 probe for amplification of a 166-bp segment of repetitive nuclear DNA by real-time polymerase chain reaction (PCR) as previously described.^31,32^ Samples with cycling threshold (Ct) values less than 34 were run on a confirmatory PCR, amplifying a 330-bp region of kinetoplast DNA using *T. cruzi* 121/122 primers.^33,34^ Amplicons were visualized on 1.5% agarose gels, and samples that yielded a band of the appropriate size were interpreted as positive in our analyses. To determine the discrete taxonomic units (DTUs) of the positive samples, a multiplex quantitative, real-time PCR was used based on amplification of the nuclear spliced leader intergenic region^35^ as previously described.^15,19^

### Statistical analysis.

To evaluate the relationship between potential risk factors and the serostatus of dogs, we performed bivariable analysis and logistic regression using the program R 1.0.136.^36^ Variables included task force (Federal Protective Services, U.S. Coast Guard, Secret Service, TSA, or two task forces within the CBP: Border Patrol or Port of Entry, or dogs in training), job/type of detection (agriculture, currency/firearms, human/narcotics, track and trail, search and rescue/cadaver, or explosives), sleeping location (indoors or outdoors), sex, age, breed, and if the location the dog worked was within triatomine range or outside it.^37^ The triatomine range was assigned on a state-level based on the CDC distribution map.^37^ Because of the small sample sizes of breeds other than Belgian Malinois, remaining breeds were combined into breed groups as follows: shepherd (German shepherd, Dutch shepherd, Belgian shepherd, Belgian Tervuren, Bohemian shepherd, Czech shepherd, Groenendael, and Sable shepherd), retriever (Labrador retriever and flat-coated retriever), pointer (German shorthaired pointer, German wirehaired pointer, and vizsla), and other (beagle, springer spaniel, and Weimaraner). Bivariable analysis using the chi-squared or Fisher’s exact test was used to identify putative risk factors, and age was analyzed using a *t*-test. Factors with a *P* ≤ 0.25 from the initial screening were used in a logistic regression model, while controlling for task force as a random effect. Generalized linear mixed models were calculated including odds ratios (ORs) and 95% confidence intervals, and factors with a *P* < 0.05 were considered significant. To determine variation in serostatus across task force, a logistic regression model was used, in which dogs in training (sampled at a training school) served as the referent to which all five management areas were compared.

## RESULTS

**Figure 1.**
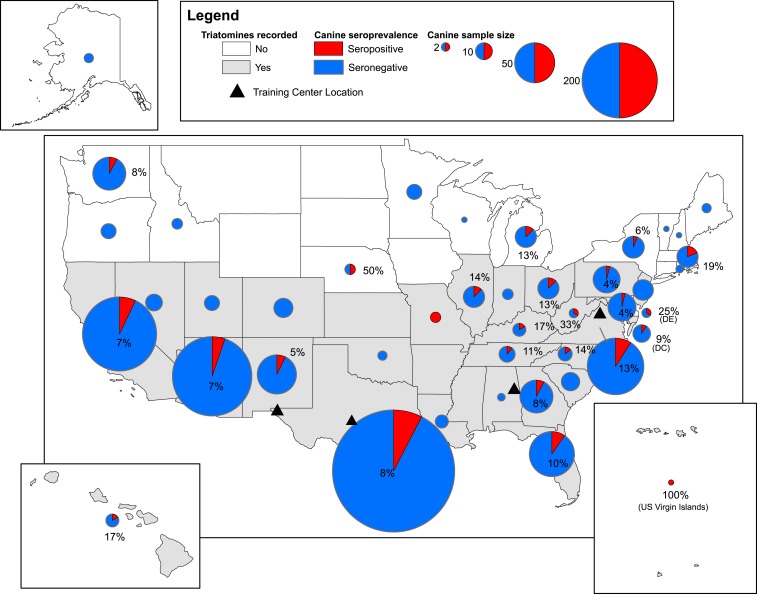
Seroprevalence of antibodies to *Trypanosoma cruzi* in the Department of Homeland Security dogs across the United States. Circles are proportional to the sample size, and red represents the percent of seropositive dogs. Canines were sampled from six different task forces and two training centers; all dogs were trained in southern United States at one of four training centers indicated by a triangle. Gray states represent the geographic range of the kissing bugs as reported by the CDC https://www.cdc.gov/parasites/chagas/. Map was created using ArcGIS, with a U.S. base layer of U.S. states and the Virgin Islands downloaded from www.census.gov. This figure appears in color at www.ajtmh.org.

A total of 1,610 DHS working dogs were sampled from across the United States from six tasks forces and two training locations, comprising approximately half of the dog workforce. Of these, 498 dogs were sampled in 2015–2016^19^ and 1,112 were sampled in 2017–2018. Dogs came from 41 states plus Washington D.C. and the U.S. Virgin Islands; one-third (33.2%) of the dogs came from Texas (Figure 1). Overall, one to 534 dogs were sampled from each state/location, with a median of eight dogs. The greatest number of dogs sampled was from the Border Patrol (32.9%), followed by the TSA (30.2%), Port of Entry dogs (21.2%), Secret Service (2.0%), Federal Protective Services (1.4%) and U.S. Coast Guard (0.87%). Finally, 11.4% of dogs were sampled while in training (training facilities in El Paso, TX, or Front Royal, VA). Most dogs sampled (58.9%) were human/narcotic detection dogs or explosive detection dogs (34.4%). Of the 1,111 dogs for which sleeping location was known, 71.5% slept indoors and 28.5% slept outdoors. There were 1,110 males (68.9%) and 500 females (31.1%). Age ranged from approximately 6 months to 13 years and 8 months, with a median of 4.4 and a mean of 4.8. The most common breed was Belgian Malinois (*n* = 583) followed by German shepherds (*n* = 489), Labrador retriever (*n* = 254), German shorthaired pointer (*n* = 147), and Dutch shepherds (*n* = 74). Of the dogs sampled, 92.4% were inside the triatomine range based on state-level reports of established triatomines.

### Seroprevalence.

Of the total dogs sampled, 7.5% (120/1,610) were seropositive based on a response on two or more independent tests and counting faint bands on the immunochromatographic tests as negative. In the bivariable analysis, *T. cruzi* serostatus was significantly different across task force (*P* = 0.013; Table 1). Logistic regression demonstrated that dog task force was not significantly associated with serostatus (OR: 3.19, 95% CI: 0.82–10.28, *P* = 0.065). Logistic regression showed a significant association between the breed and serostatus after controlling for task force as a random effect (OR: 2.21, 95% CI: 1.12–4.10, *P* = 0.01), in which retrievers were associated with a significantly higher seroprevalence (12.0%) than Belgian Malinois (7.2%, referent). Serostatus did not vary significantly by sex, age, detection type/job, sleeping inside/outside, or location within or outside the triatomine range.

**Table 1:** Results of bivariable analysis of potential risk factors for *Trypanosoma cruzi* exposure among the Department of Homeland Security working dogs across the United States

Variable		Sample size, no. (%)	Seronegative, no. (%)	Seropositive, no. (%)	*P*-value
Sex	Male	1,110 (68.9)	1,029 (92.7)	81 (7.3)	0.80
	Female	500 (31.1)	461 (92.2)	39 (7.8)	
Task force	Border Patrol	530 (32.9)	480 (90.6)	50 (9.4)	0.013*
	Coast Guard	14 (0.87)	12 (85.7)	2 (14.3)	
	Federal Protective Services	22 (1.4)	18 (81.8)	4 (18.2)	
	Port of Entry	342 (21.2)	329 (96.2)	13 (3.8)	
	Secret Service	32 (2.0)	29 (90.6)	3 (9.4)	
	Transportation Security Administration	486 (30.2)	450 (92.6)	36 (7.4)	
	Training	184 (11.4)	172 (93.5)	12 (6.5)	
Breed group	Shepherd	576 (35.8)	539 (93.6)	37 (6.4)	0.054*
	Belgian Malinois	583 (36.2)	541 (92.8)	42 (7.2)	
	Pointer	174 (10.8)	165 (94.8)	9 (5.2)	
	Retriever	258 (16.1)	227 (88.0)	31 (12.0)	
	Other	19 (1.2)	18 (94.7)	1 (5.3)	
Detection	Agriculture	25 (1.6)	23 (92.0)	2 (8.0)	0.89*
	Currency/firearms	22 (1.4)	20 (90.9)	2 (9.1)	
	Human/narcotics	949 (58.9)	883 (93.0)	66 (8.6)	
	Track and trail	35 (2.2)	32 (91.4)	3 (8.6)	
	Search and rescue/cadaver	25 (1.6)	23 (92.0)	2 (8.0)	
	Explosives	554 (34.4)	509 (91.9)	45 (8.1)	
Range	Inside triatomine range	1,488 (92.4)	1,379 (92.7)	109 (7.3)	0.61
	Outside triatomine range	120 (7.5)	109 (90.8)	11 (9.2)	
Sleep	Indoors	794 (49.3)	739 (93.1)	55 (6.9)	0.55
	Outdoors	317 (19.7)	289 (91.2)	28 (8.8)	
	Unknown	499 (31.0)	462 (92.6)	37 (7.4)	
	Average age†		4.8	5.0	0.39†

* Expected cell count in the contingency table < 5; Fisher's exact test was reported instead of the chi-squared test.

† *t*-test performed instead of the chi-squared test.

Twenty-eight of the dogs sampled in 2015–2016 that were in training at that time were resampled in 2017–2018 after being deployed to a task force; all were working in the Border Patrol or at Ports of Entry in 2017–2018. Of these 28 dogs, two were positive during training in 2015–2016 on two or more independent serological assays. One of these dogs remained positive during the 2017–2018 sampling, whereas the other was positive on both rapid tests in April 2016 yet had inconclusive test results (faint bands) in June 2017 and was, therefore, considered negative in the current analysis.

#### Molecular detection of parasite DNA and *T. cruzi* strain types.

*Trypanosoma cruzi* DNA was detected in the buffy coat fraction of the blood in four of 1,610 (0.25%) dog samples using both a screening and confirmatory assay. In addition, there were three dogs with samples that amplified in the screening PCR with a Ct value of 31, yet these samples were negative on the subsequent assay and, therefore, considered negative. Three of the PCR-positive dogs were sampled only in 2015–2016 and were from Texas: one dog had a Ct value of 33.5 and DTU TcIV, another had a Ct of 30.3 and was coinfected with TcI/TcIV, and one had a Ct value of 33.1 and was untypable, as previously reported.^19^ The remaining PCR-positive dog was a 3-year-old female Labrador retriever that worked for Amtrak to perform explosives detection; this dog was working in Washington D.C. at the time of sampling and lived in Arlington, Virginia, with her handler. She was reported to sleep indoors and was sampled in June 2017, at which time she tested positive for antibodies by all three serology assays with a high titer (1,280) on IFA. This dog had a Ct of 26.1, and using the multiplex real-time PCR to determine *T. cruzi* DTUs, we found that this dog harbored DTU TcIV.

## DISCUSSION

*Trypanosoma cruzi* transmission in the United States was first reported in dogs in 1972,^38^ and locally acquired human infections were first recognized in 1955,^39^although triatomines have been recognized from human dwellings since 1930s.^40,41^ Infection in dogs has been reported from at least eight southern states including Texas, Louisiana, Oklahoma, Tennessee, Virginia, California, Georgia, and South Carolina.^13,16,28,42–48^ Herein, we tested 1,610 dogs for *T. cruzi* exposure from 41 states as well as Washington D.C. and the U.S. Virgin Islands. To our knowledge, this is the largest domestic dog serosurvey for *T. cruzi* antibodies performed in the United States and the first to include dogs from 41 states.

We found that working dogs had widespread exposure to *T. cruzi* across the United States. Overall, we found that seroprevalence in DHS working dogs across the United States was 7.5%. Many dogs had inconclusive results (faint bands) on Stat-Pak^®^ or Chagas Detect^TM^ and were considered negative for our analysis. However, if our criteria for categorizing a dog as positive in this study were more inclusive—still requiring positive reactions on two independent serologic testing platforms, yet allowing for the inclusion of such faint bands as a positive result—the seroprevalence could be as high as 23.1%. The degree to which false-positive reactions influence the apparent seroprevalence estimate is unknown. Of the three serology tests, the IFA uses the whole antigenic fractions of the *T. cruzi* epimastigote, which can allow for nonspecific reactions (false positives) with related parasites (e.g., Leishmania). The two immunochromatographic assays use recombinant antigens, and high specificity has been reported in previous studies of humans and dogs (94–99.5%).^49,50^ However, in the absence of gold standard testing methods, the definitive identification of false positives and calculation of specificity pose challenges. To try to account for imperfect test diagnostics, we used up to three independent assays and required positivity on at least two to classify a sample as positive; furthermore, very faint bands on the rapid tests (inconclusive results) did not count toward the criterion of positivity. Nonetheless, the imperfections in canine Chagas diagnostics may account for unquantified levels of the misclassification bias in our sample set.

Surprisingly, we found no significant difference between seroprevalence of dogs within versus outside the triatomine range (*P* = 0.61), with 9.2% (*n* = 120) and 7.3% (*n* = 1,488) of dogs seropositive, respectively. The 11 seropositive dogs residing in Washington (3), Massachusetts (3), Michigan (2), Nebraska (2), and New York (1) outside the kissing bug range likely demonstrate movement of *T. cruzi*–exposed dogs from locations where transmission naturally occurs. Given that DHS working dogs train at one of four centers in the southern Unites States—all in states with established triatomine populations—training may represent an at-risk time for exposure. We followed up on the procurement and training histories of the 11 seropositive dogs that were found to be outside the kissing bug range and found that all had spent at least some time training or living in the south where local transmission could have occurred. Individual dogs can move for various reasons including owner relocation, travel, and adoption programs; these movements allow for the translocation of infections that might not be acquired or transmitted in the new environment, as has been described for other vector-borne diseases, such as heartworm, Lyme disease, ehrlichiosis, and anaplasmosis.^51,52^ These data demonstrate the need for heightened veterinary awareness for infection with vector-borne diseases in dogs outside endemic areas. Furthermore, in dogs with heart disease, knowledge of a travel history to a southern state with endemic triatomines may raise the index of suspicion for Chagas disease.

Antibodies to *T. cruzi* have been found in 48 different dog breeds in the United States.^44^ We found that the retriever breed group was associated with a significantly higher seroprevalence (12.0%) than Belgian Malinois (7.2%, *P* = 0.01), which served as a referent in the analysis. Similar to our findings in retrievers, a retrospective study of serologically and/or histopathologically *T. cruzi*–positive dogs in Texas found that sporting breeds—primarily made up of Labrador retrievers and English pointers—made up 51.6% of the cases, compared with 8.1% of their cases being herding dogs (which includes Belgian Malinois and German shepherds).^44^ The high seroprevalence seen in retrievers could be due to the difference in life history before training at DHS facilities. This could include the difference in housing (indoor/outdoor kennel), geographic location of kennel, or an individual propensity for consuming bugs, rather than breed predilection. The retrievers are more likely to be bred in the United States, whereas most of the Belgian Malinois are bred in Europe. Seroprevalence was higher in dogs in the Federal Protective Services (18.2%) than dogs at the training center (6.5%) but was not significant (*P* = 0.065). The dogs serving in the Federal Protective Services are the only dogs that train at a facility in Alabama—their higher exposure could be due to exposure at that facility or differences in procurement before training as Federal Protective Service dogs.

Although previous studies of *T. cruzi* infection in dogs found that exposure increased with age,^11,15,19,22,28^ owing to older dogs having a longer time for exposure to *T. cruzi*, we observed no statistical difference in ages of exposed versus unexposed dogs (*P* = 0.39). In addition, previous studies in Texas, Tennessee, and Louisiana have concluded that dogs sleeping outdoors have greater exposure to the parasite,^14,17,19,28^ yet the working dogs showed no difference in exposure based on sleeping location (*P* = 0.55). The unique life histories of these working dogs, which include months of training outside early in life, may account for different transmission environments compared with other naturally exposed dogs.

Twenty-eight dogs were tested while in training during our prior study,^19^ then again after deployment to their task force one to 2 years later in the current study. Twenty-six dogs were negative at both time points, and one dog was consistently positive at both time points. One dog, however, was associated with different test results between the years. This dog was in training in El Paso, TX, before being deployed to San Ysidro, CA. The dog status changed from being positive on both immunochromatographic assays in 2016 to having inconclusive reactions (faint bands) on these assays in 2017, which were interpreted as negative; during both years of testing, the dog was IFA negative. Discordant test results are common in *T. cruzi* diagnostics, and testing is limited by a lack of a gold standard^17,19^; the use of multiple serology assays is widely used to assign positivity.^14,15,19,24,27,29,53^ Although *T. cruzi* infections are commonly thought to be lifelong in the absence of antiparasitic treatments, spontaneous seroreversion has been documented in mice, humans, and dogs^9,11,54–58^; therefore, it is possible that this dog seroreverted. Alternatively, the two assays could be inaccurate because of test cross-reactions or other reasons for a false-positive result. A need for improved diagnostics for both veterinary epidemiological research and individual diagnoses is critical to allow for improved estimation of infection prevalence and allow for earlier detection which could improve prognosis.

Only four dogs were positive for *T. cruzi* DNA circulating in their blood by the test criteria, which required positive PCR results on two independent assays, although an additional three dogs were positive only on the screening PCR. The low rate of PCR positivity (0.25%) relative to the detected seropositivity in our study (7.5%) was not an unexpected finding. Similarly, in field studies of other dog populations, the seropositivity rate greatly exceeded the PCR-positivity rate.^14,16^ By contrast, however, the frequency of PCR-positive dogs was greater than seropositive dogs in animal shelters in Louisiana.^59^ Factors that may contribute to the low frequency of PCR positivity include short duration of parasitemia (3- to 6-week window following initial infection) in experimentally infected dogs,^60,61^ and blood sampling across the year, which does not always coincide with peak triatomine activity when acute infections are expected to be most common. Additionally, our sampling likely also included chronically infected dogs that may have been infected years before and, in some cases, are now living in areas with no entomologic risk. All PCR-positive dogs were residing within the kissing bug range—three were in Texas (DTUs TcI and TcI/TcIV mix) and one was from Washington D.C. (DTU TcIV). TcI and TcIV have been commonly found in wildlife hosts and vectors in Texas.^4,15,19^ Previous strain typing in dogs in the United States has commonly found DTU TcIV,^62,63^ although coinfections of TcI/TcIV have been found,^15,19,63^ and one study in south Texas found exclusively TcI.^64^ This variation in DTU could be driven by the vector that the dogs are exposed to because geographic distribution varies by triatomine species and there are associations between the vector and strain type.^65^ The dog from Washington D.C. was bred in Texas and resided there until she was 7 months old in April 2015, at which time she was purchased by the DHS and transferred to Alabama for training. She trained in Alabama until January 2016, then transferred to Washington D.C. where she resided until testing. The only travel was to Alabama for a week of training in February 2017. This dog worked in Washington D.C., but lived with the handler in Arlington, Virginia. Although there are no prior reports of Chagas disease in dogs in Washington D.C., infection with *T. cruzi* has been reported in wildlife and dogs in the neighboring state of Virginia,^66–68^ including a finding of TcIV in a cocker spaniel from Virginia.^62^ In addition, the Kissing Bug Citizen Science Program, run by Texas A&M University, has received multiple specimens of triatomines from Virginia and surrounding areas, including insects infected with TcIV.^65^ *Triatoma sanguisuga* is the primary vector in this area and is more likely to be infected with TcIV.^65^ An infection prevalence of 33% (*n* = 464) was found in raccoons (*Procyon lotor*) in urban/suburban areas outside D.C.,^66^ which have previously been shown to be primarily infected with TcIV.^1,4,63^ It is possible that this dog was infected locally because TcIV has been found to be circulating in the local wildlife. Understanding of *T. cruzi* strain types circulating in dogs is important because different strain types are potentially associated with different clinical outcomes.

Determining the prevalence of *T. cruzi* in dogs has practical implications for disease risk management and could assist with improved control measures. These findings should raise awareness among medical practitioners regarding *T. cruzi* infection throughout the United States. Furthermore, understanding the distribution and risk factors for zoonotic parasite infection in natural populations of dogs could potentially be informative for human health.
